# Deficiency in Toll-interacting protein (Tollip) skews inflamed yet incompetent innate leukocytes *in vivo* during DSS-induced septic colitis

**DOI:** 10.1038/srep34672

**Published:** 2016-10-05

**Authors:** Na Diao, Yao Zhang, Keqiang Chen, Ruoxi Yuan, Christina Lee, Shuo Geng, Elizabeth Kowalski, Wen Guo, Huabao Xiong, Mingsong Li, Liwu Li

**Affiliations:** 1 Department of Biological Sciences, Biomedical Engineering, Medicine, Virginia Tech, 24061 USA; 2 Guangdong Provincial Key Laboratory of Gastroenterology, Department of Gastroenterology, Nanfang Hospital, Southern Medical University, 510515 People’s Republic of China.; 3 Department of Medicine, Immunology Institute, Icahn School of Medicine at Mount Sinai, New York, NY 10029, USA

## Abstract

Functionally compromised neutrophils contribute to adverse clinical outcomes in patients with severe inflammation and injury such as colitis and sepsis. However, the ontogeny of dysfunctional neutrophil during septic colitis remain poorly understood. We report that the dysfunctional neutrophil may be derived by the suppression of Toll-interacting-protein (Tollip). We observed that Tollip deficient neutrophils had compromised migratory capacity toward bacterial product fMLF due to reduced activity of AKT and reduction of FPR2, reduced potential to generate bacterial-killing neutrophil extra-cellular trap (NET), and compromised bacterial killing activity. On the other hand, Tollip deficient neutrophils had elevated levels of CCR5, responsible for their homing to sterile inflamed tissues. The inflamed and incompetent neutrophil phenotype was also observed *in vivo* in Tollip deficient mice subjected to DSS-induced colitis. We observed that TUDCA, a compound capable of restoring Tollip cellular function, can potently alleviate the severity of DSS-induced colitis. In humans, we observed significantly reduced Tollip levels in peripheral blood collected from human colitis patients as compared to blood samples from healthy donors. Collectively, our data reveal a novel mechanism in Tollip alteration that underlies the inflamed and incompetent polarization of neutrophils leading to severe outcomes of colitis.

Proper regulation of neutrophil function is essential for anti-microbial defense and immune homeostasis. Normal neutrophils in healthy individuals are capable of balanced functions such as chemotaxis, degranulation, and the generation of extra-cellular trap (NET)^1^,^2^,^3^,^4^. In sharp contrast, compromised chemotaxis toward bacterial sources and reduced NET generation represent two of the most striking alterations of blood neutrophils from human septic patients as well as septic animal models^5^,^6^. Human septic neutrophils exhibit so-called selective “migratory paralysis” and fail to properly migrate toward microbial chemo-attractants such as fMLF^3^. Complementing this observation, FPR2 (fMLF receptor) knockout mice were shown to have exacerbated experimental sepsis^7^. On the other hand, septic human neutrophils tend to express higher levels of CCR5, a chemokine receptor for host sterile chemokines that attract neutrophils to sterile inflammed host tissue^8^,^9^. This may correlate with host tissue inflammation and co-lateral multi-organ damage. Furthermore, septic neutrophils have compromised potential to form NET, a vital anti-microbial defense mechanism to clear disseminated microorganisms^2^,^6^. Despite the well-known incompetent phenotype of paralytic neutrophils in both human septic patients and experimental septic animals, the underlying mechanisms are poorly understood.

Toll-interacting-protein (Tollip) is a homeostatic regulator in innate leukocytes responsible for suppressing the pro-inflammatory polarization of macrophages^10^. Our previous study demonstrates that Tollip fulfils its homeostatic function through facilitating autophagy completion^11^. Suppression of Tollip through reduced expression or altered cellular localization may lead to disrupted autophagy, cellular stress and inflammation^11^. We also demonstrated that Tauroursodeoxycholic Acid (TUDCA), a potent compound in relieving cellular stress, can effectively enhance the homeostatic function of Tollip through maintaining its proper lysosome localization and expression^11^. Despite these intriguing studies, the potential roles of Tollip in neutrophils remain unknown. Given the emerging roles of autophagy completion in the resolution of inflammation as well as NET formation in neutrophils^12^, we aim to test whether Tollip deletion may also compromise the resolution of neutrophil inflammation and NET forming potential.

Oral administration of Dextran Sulfate Sodium (DSS) in the murine model serves as a classical model of acute gastrointestinal leakage and septic injury^13^. In this study, we utilized the murine DSS septic colitis model to test the hypothesis that Tollip deficiency may exacerbate DSS colitis through polarizing “inflamed yet incompetent” neutrophils, inciting tissue inflammation, and compromising host anti-microbial defense. The underlying mechanisms were examined by using wild type (WT) and Tollip deficient neutrophils *in vitro*. Furthermore, we tested whether TUDCA may alleviate DSS-induced murine colitis. The levels of Tollip in human blood neutrophils collected from patients with acute colitis were also determined.

## Results

### Tollip deficiency increased colonic mucosal damage in DSS induced colitis

**Figure 1:**
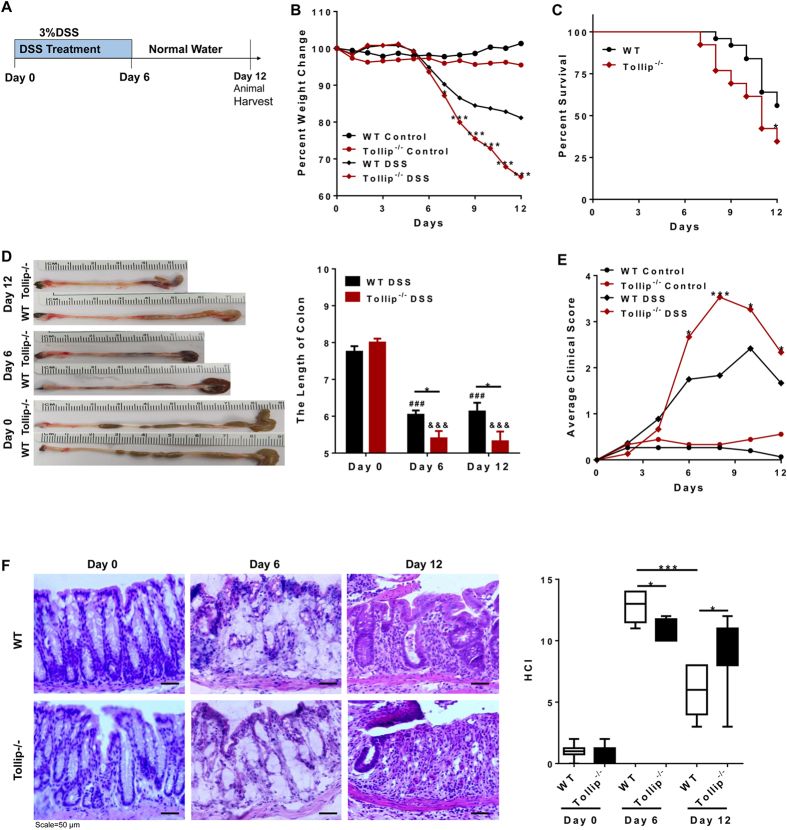
Tollip^−/−^ mice are more susceptible to DSS induced acute colon injury. (**A)** Schematic of the acute model of DSS-induced ulcerative colitis. (**B)** Weight loss of WT, Tollip^−/−^ naïve mice and DSS-treated WT, Tollip^−/−^ mice. (**C)** The survive curve. **(D)** Length and gross morphology of colon. (**E)** Average clinical scores (weight loss, stool consistency, bleeding) of indicated mice. (**F)** Representative H&E staining in colon tissues from WT and Tollip^−/−^ mice at day 0, day 6, day 12 after subjected to DSS, and, histology assessment according to criteria in Supplementary Fig. 2. (DSS-treated WT, n = 25; DSS-treated Tollip^−/−^, n = 26). The symbol *,***indicated P < 0.05, P < 0.001 between the DSS-treated gene-deficient strains compared with DSS-treated WT. The symbols ^##^,^###^indicated P < 0.01, P < 0.001 and ^&&&^P < 0.001, between the WT control and DSS-treated WT or between Tollip^−/−^ control and DSS-treated Tollip^−/−^ mice, respectively. WT control, n = 7; Tollip^−/−^ control, n = 7; DSS-treated WT, n = 22; DSS-treated Tollip^−/−^, n = 15. Error bars have been omitted from the weight loss data and clinical score data for clarity of presentation.

In order to develop a system to examine the role of Tollip in the polarization of innate leukocytes *in vivo*, we utilized the well-described DSS-induced colon inflammation and septic colitis model. We re-capitulated the finding that Tollip deficient mice developed severe colitis when administered with DSS^14^ (Fig. 1A)^14^. As shown in Fig. 1, Tollip-deficient mice displayed a rapid and significant loss in body weight (Fig. 1B), higher mortality (Fig. 1C), shorter colon length (Fig. 1D), and overall higher clinical features associated with gastrointestinal disease (Fig. 1E, Supplementary Fig. S1) as compared to WT mice following DSS challenge. Further histological analysis revealed that the Tollip deficient mice had more histological damage (cellular infiltration, goblet cell depletion, distortion/damage to crypt architecture) as compared to wild type mice. (Fig. 1F, and supplementary Fig. S2). Taken together, these data recapitulated an acute inflammatory model system to further study the role of Tollip during the *in vivo* polarization of innate leukocytes.

### Tollip deficiency drives an inflamed yet incompetent neutrophil phenotype *in vivo*

**Figure 2:**
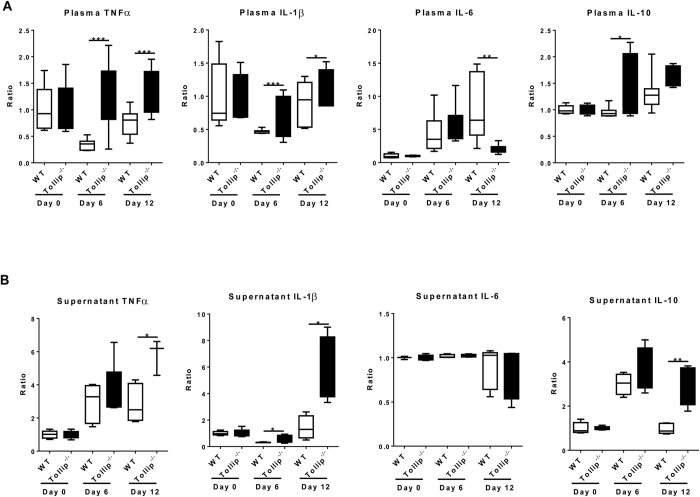
Tollip^−/−^ mice showed enhanced pro-inflammatory response both on systemic and local level after challenged with DSS. (**A,B**) TNT-a, IL-1β, IL-6 and IL-10 levels in plasma and colon culture supernatant from WT and Tollip deficient mice. WT control *n* = 5; Tollip^−/−^ control *n* = 7; DSS treated WT (day 6), *n* = 7; DSS treated Tollip^−/−^ (day 6), *n* = 7; DSS treated WT (day 12), *n* = 7; DSS treated Tollip^−/−^ (day 12), *n* = 4. *P < 0.05, ** P < 0.01, *** P < 0.01.

As the goal of this study is to define the role of Tollip during the inflammatory polarization of innate leukocytes *in vivo*, we further utilized the DSS-induced inflammation and colitis model to examine the inflammatory parameters in WT and Tollip deficient mice. First, we examined the expression profiles of selected pro-inflammatory cytokines in circulating blood. As classically noted, wild type leukocytes or animals exhibit a compensatory “tolerance” phenotype following a severe challenge with microbes or microbial products Lipopolysaccharide (LPS). The tolerant cells or animals had reduced circulating levels of inflammatory mediators such as TNFα and IL-1β^15^. Indeed, we observed that the plasma circulating levels of TNFα were significantly suppressed following 6 days of DSS challenged (Fig. 2A). Upon stoppage of DSS challenge, the tolerance phenotype disappears and the plasma levels of TNFα returned back to resting levels. In sharp contrast, we noticed that Tollip deficient mice did not possess tolerance, as reflected in the constitutively higher levels of TNFα and IL-1β following 6 days of DSS (Fig. 2A). We further examined the local gut mucosal secretion of TNFα and IL-1β, and observed prolonged elevation of selected cytokines within the mucosal tissues of Tollip deficient mice as compared to WT mice (Fig. 2B). These data are consistent with the notion that Tollip serves as a negative regulator of the inflammatory mediators during severe and acute inflammation^10^,^15^.

Despite the known role of Tollip in macrophages and monocytes^16^,^17^, its role in the regulation of neutrophils remain unknown. Next, we selectively examined the neutrophil phenotype comparing WT and Tollip deficient mice. Neutrophils are capable of inflammatory and modulatory functions through the expression of distinct subsets of chemokine receptors such as CCR5 and FPR2^8^,^18^. Under acute septic condition, neutrophils in both humans and mice were shown to have a skewed expression profile of CCR5 and FPR2, with elevated CCR5 and reduced FPR2 levels^7^,^19^. As a consequence, septic neutrophils are skewed toward sterile tissue sites due to elevated CCR5 levels, and failed to home toward bacterial infection sites due to reduced FPR2 levels. Such “inflamed” yet “incompetent” neutrophil may subject the host to high morbidity and mortality associated with septic insult^5^. Given the elevated inflammatory phenotype in Tollip deficient mice challenged with DSS, we tested the hypothesis that Tollip may be critically involved in the polarization of neutrophils toward the inflamed yet incompetent state (N_*ii*_).

**Figure 3:**
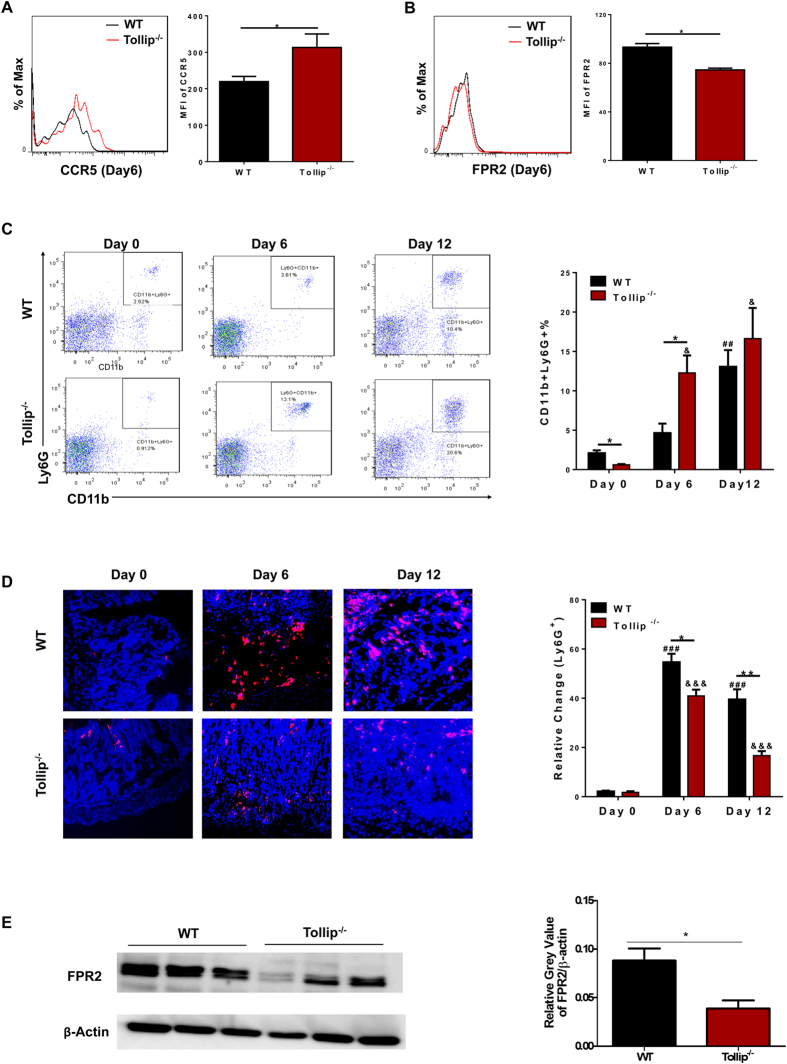
Neutrophils in Tollip^−/−^ mice accumulated in blood and failed to migration to injured mucosa after exposure to DSS. (**A,B)** CCR5 and FPR2 expression on neutrophils (gated on Ly6G + CD11b + ) in blood via flow cytometry. (**C)** Flow cytometry about neutrophils (CD11b^+^ Ly6G^+^) in blood harvested at day 0, day 6, day12 during the process of experimental colitis. (**D)** Immuno-histological staining (left) of neutrophils (Ly6G^+^) in the injured mucosa of colon; assessment (right) about neutrophils with stained particles using Image J. (**E**) Western blot of FPR2 expression on purified neutrophils in blood at day 6. The symbols *,**indicate P < 0.05, 0.01 between the DSS-treated gene-deficient strains compared with DSS-treated WT. The symbols ^#^,^##^,^###^indicate P < 0.05, 0.01, 0.001, and ^&^,^&&&^indicate P < 0.05, 0.001 between the WT control and DSS-treated WT or between Tollip^−/−^ control and DSS-treated Tollip^−/−^ mice, respectively. WT control *n* = 3; Tollip^−/−^ control *n* = 3; DSS treated WT (day 6), *n* = 5; DSS treated Tollip^−/−^ (day 6), *n* = 5; DSS treated WT (day 12), *n* = 5; DSS treated Tollip^−/−^ (day 12), *n* = 4.

Through flow cytometry analyses, we observed that blood neutrophils in Tollip deficient mice challenged with DSS expressed significantly higher levels of CCR5 and reduced levels of FPR2 as compared to WT mice (Fig. 3A,B). We further tested the cellular population of neutrophils in blood leukocytes, and observed correspondingly higher percentage of blood neutrophils in Tollip deficient mice challenged with DSS as compared to WT mice (Fig. 3C). In sharp contrast, we observed significantly reduced numbers of neutrophils in the gut lesion areas of Tollip deficient mice as compared to WT mice challenged with DSS (Fig. 3D). The reduced infiltration of neutrophils to intestinal lesion sites with gut microflora correlated with reduced expression of neutrophil FPR2 (Fig. 3B). We observed similarly skewed monocytes with elevated expression of CCR5 in the gut as well as circulating blood of Tollip^−/−^ mice as compared to WT mice (Supplementary Fig. S3). Together, our data suggest that neutrophils may be skewed toward an incompetent and inflamed state in Tollip deficient mice challenged with DSS.

### Tollip deficiency drives an inflamed yet incompetent neutrophil phenotype *in vitro*

**Figure 4:**
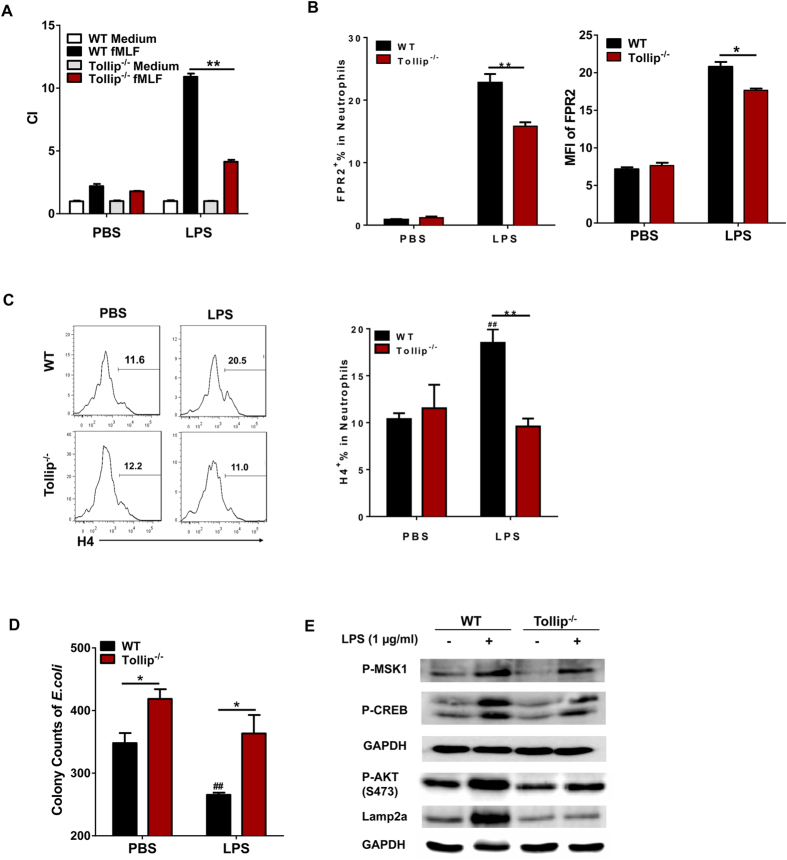
Tollip^−/−^ neutrophils demonstrated an inflamed yet incompetent phenotype *in vitro*. (**A)** Chemotaxis of neutrophils from bone marrow to fMLF (10^−5^mM). (**B)** FPR2 expression on neutrophils (gated on Ly6G + CD11b + ) from bone marrow via flow cytometry. (**C)** H4 expression in neutrophils (gated on Ly6G + CD11b + ). (**D)** Neutrophil bacteria killing assay. (**E)** Western blot of purified neutrophils from bone marrow. The symbols *,**indicate P < 0.05, 0.01 between the DSS-treated gene-deficient strains compared with DSS-treated WT. The symbols ^#^,^##^indicate P < 0.05, 0.01, between the WT control and DSS-treated WT.

Given the skewing of incompetent neutrophils in Tollip deficient mice, we further characterized the modulation of Tollip deficient neutrophils through *in vitro* culture, in terms of their migratory capability, as well as bacterial killing activity. We first tested the migratory ability of neutrophils toward FPR1/2 ligand fMLF through employing the *in vitro* migration assay. As shown in Fig. 4A, we observed a robust induction of neutrophil migration toward fMLF with WT neutrophils stimulated with high dose of LPS, an agent known to induce the potent expression of FPR1/2. In sharp contrast, LPS-induced neutrophil migration toward fMLF with Tollip deficient neutrophils were significantly compromised as compared to the WT neutrophils. We further performed flow cytometry analyses of cell surface FPR2 levels and observed reduced induction of FPR2 on Tollip deficient neutrophils as compared to WT neutrophils (Fig. 4B). Our data complement our *in vivo* data and support the conclusion that Tollip is involved in the induction of FPR2 expression in neutrophils, and that the lack of Tollip jeopardizes the migratory competency of neutrophils toward bacterial cues.

Furthermore, we tested the generation of neutrophil extra-cellular trap (NET) in WT and Tollip deficient neutrophils. Neutrophil NET is another critical mechanism for host protection against infection and clearance of potentially harmful inflammatory debris^2^,^6^. We observed that WT neutrophils can be induced to generate NET when stimulated with high dose LPS (Fig. 4C). In contrast, the induction of NET in Tollip deficient neutrophils was significantly impaired as compared to WT neutrophils (Fig. 4C). Consequently, we observed significantly compromised bacterial killing capacity of Tollip deficient neutrophils as compared to WT neutrophils (Fig. 4D).

Despite the compromised function of Tollip deficient neutrophils in migration toward fMLF and NET generation, we observed increased inflammatory secretion of myeloperoxidase from Tollip deficient neutrophils stimulated with high dose LPS as compared to WT neutrophil, further supporting the notion that Tollip deficiency may yield the inflamed yet incompetent neutrophil phenotype resembling septic neutrophils (Supplementary Fig. S4).

### Deficiency in Tollip compromises the activation of PI3K pathway responsible for the expression of FPR2 and generation of NET

Neutrophil FPR2 expression has been shown to be controlled by SP1 and CREB with upstream activating kinases such as PI3K, ERK and MSK1^18^,^20^. Next, we tested whether Tollip may regulate the expression of FPR2 in neutrophils through modulating the activation of AKT. Indeed, we observed that the levels of p-CREB were significantly reduced in Tollip^−/−^ neutrophils as compared to WT neutrophils with or without LPS treatment (Fig. 4E). In addition, the phosphorylated levels of AKT and MSK1 were reduced in Tollip^−/−^ neutrophils as compared to WT neutrophils (Fig. 4E). Since the phosphorylation of AKT may be counter-acted by the AKT selective phosphatase PHLPP^21^, we probed for the levels of PHLPP and observed higher induction of PHLPP by LPS in Tollip^−/−^ neutrophils (Fig. 4E).

### TUDCA, a compound capable of restoring Tollip function, alleviates DSS-induced colitis *in vivo*

**Figure 5:**
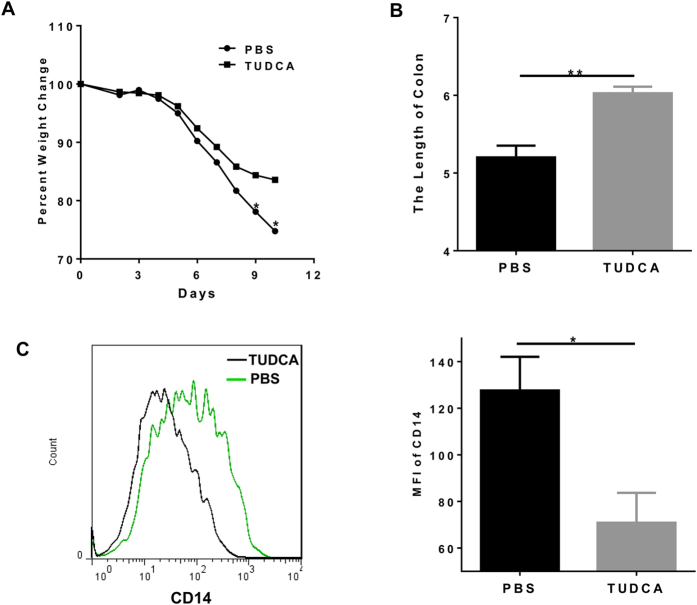
TUDCA alleviates DSS-induced colitis. (**A)** Weight loss of DSS-treated WT mice with TUDCA treatment or PBS as control. Each group, *n* = 10. (**B)** Length and gross morphology of colon. (**C**) CD14 expression on neutrophils in blood (gated on Ly6G + CD11b + ). *P < 0.05, ** P < 0.01.

We previously reported that the proper localization of Tollip at lysosome is critical to maintain the cellular protective effect of Tollip, and that Tauroursodeoxycholic Acid (TUDCA) can potently restore Tollip subcellular localization and function^11^. Based on this mechanistic finding, we further tested whether TUDCA may alleviate DSS-induced colitis. Indeed, daily administration of TUDCA significantly alleviated body weight loss (Fig. 5A) and colon shrinkage (Fig. 5B) caused by DSS. Furthermore, TUDCA significantly reduced the inflammatory activation of circulating neutrophils as measured by the surface levels of CD14 (Fig. 5C).

### Tollip expression is reduced in human neutrophils from patients with colitis

**Figure 6:**
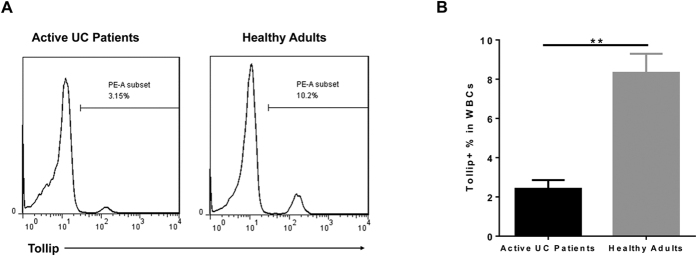
Tollip expression is reduced in human neutrophils from patients with colitis. (**A)** Representative flow cytometry results of Tollip staining in white blood cells. (**B**) statistical analysis of the percentages of Tollip + cells in white blood cells. Healthy adults, n = 8; active UC patients, n = 6. ** P < 0.01.

Given the significant functional role of Tollip during neutrophil polarization and colitis, we further tested whether Tollip expression may be altered in human patients with colitis. Although a previous study studied the Tollip levels in colonic epithelial cells from patients with inflammatory bowel disease, the data were inconclusive^22^. Furthermore, no data is currently available to address the regulation of Tollip in neutrophils. We have collected human peripheral blood mononuclear cells (PBMC) comparing healthy donors and acute colitis patients. As shown in Fig. 6, we observed a significant reduction in the levels of Tollip in colitis patients as compared to healthy donors.

## Discussion

Our data reveal that Tollip deficiency skews the programming of inflamed yet incompetent neutrophils that underlie the pathogenesis of acute injury and septic colitis in mice and humans. Neutrophils from Tollip deficient mice subjected to DSS-induced septic colitis exhibited heightened inflammatory responses such as elevated levels CCR5, and increased secretion of MPO. On the other hand, Tollip deficient neutrophils had compromised migratory potential toward microbial product fMLF due to reduced expression of FPR2, tempered NET forming potential and reduced bacterial killing activity. We also documented that human neutrophils harvested from colitis patients had significantly reduced levels of Tollip. Restoration of Tollip function through the application of TUDCA effectively alleviated the severity of DSS-induced colitis.

Our data extend emerging studies in the area of innate immune cell polarization and memory^23^,^24^. Extensive studies reveal that monocytes/macrophages can be programmed into either a non-resolving inflammatory state or an opposite resolving homeostatic tolerance^23^,^25^,^26^. However, our understanding of neutrophil polarization is limited, despite the fact that previous clinical and basic studies have long suspected the unique polarization/adaptation of neutrophils among health and various disease conditions^27^,^28^. With particular relevance to injury and sepsis, dysfunctional neutrophils with overt inflammation and compromised bacterial killing potency have been well noticed^3^,^6^. However, mechanistic understanding has been lacking, and thus, our current study fills this pressing void. Our data complement these intriguing observations with regard to neutrophil polarization, and suggest the existence of an “inflamed and incompetent” neutrophil state due to Tollip disruption. The compromised neutrophil state is reflected in the altered migratory ability toward microbial cues, and elevated inflammatory neutrophil presence in circulation, as well as compromised NET forming potential in killing bacteria. With regard to molecular mechanisms, we identified Tollip as a key modulator for effective neutrophil function. Our data demonstrated that, during DSS-induced colitis, Tollip deficiency significantly skews neutrophils into the inflamed and incompetent state. At the mechanistic level, Tollip may assist proper neutrophil function such as FPR2 expression and NET generation through AKT activation.

Our data support the therapeutic potential of TUDCA in the treatment of colitic sepsis through augmenting the function of Tollip in neutrophils. TUDCA has also been shown to have beneficial effects to treat various chronic inflammatory diseases such as diabetes and wound healing^26^,^29^. With regard to colitis, previous studies reported that the oral administration of TUDCA may alleviate experimental colitis through relieving epithelial cell stress and preventing intestinal epithelial cell death, although these studies also suggest that TUDCA may also benefit the host survival through reducing inflammation^30^,^31^. In our previous study, we reported that TUDCA can prevent macrophage activation through restoring Tollip function during autophagy completion^11^. Complementing these studies, our current data reveal that TUDCA can reduce the inflammatory activation of neutrophils. Furthermore, in contrast to previous studies that examine the local mucosal protective effect of TUDCA through oral gavage, our current study demonstrates that the intra-peritoneal injection of TUDCA can exert a systemic protective effect on DSS-induced colitis.

Our human studies further support the conclusion that the reduction in Tollip may jeopardize proper neutrophil functions necessary for host defense toward colitis and severe inflammation. We observed reduced expression of Tollip within blood circulating neutrophils collected from colitis patients as compared to neutrophils from health donors. In contrast, a previous survey of human patients with colitis focused on studing the expression of Tollip within epithelial cells and observed no difference in Tollip expression comparing healthy colonic epithelia with colonic epithelia from colitis patients^22^.

Under severe challenges such as DSS-induced colitis, disseminated infection and sepsis, Tollip serves as a negative regulator to dampen the excessive inflammation and induce tolerance, through facilitating the activation of PI3K/AKT as well as autophagy completion^11^,^32^. PI3K/AKT has been shown to be a key pathway involved in the anti-inflammatory resolution of innate leukocytes and contributes to the development of tolerance^15^,^32^. Consistent with these previous reports, we herein demonstrated that Tollip deficient neutrophils have defective AKT activation. Consequently, we observed that Tollip deficient mice and cells have sustained elevation of TNFα and lack tolerance. In addition to the modulation of AKT activation, Tollip can down-regulate inflammatory leukocyte activation through facilitating lysosome fusion and cellular homeostasis^11^. Consistent with this observation, we observed in this study that the application of TUDCA, a compound capable of restoring lysosome fusion and cellular homeostasis, can effectively alleviate gut inflammation in mice challenged with DSS. Our current study extends the previous mechanistic studies with regard to the involvement of Tollip in modulating leukocyte inflammatory responses. It is also worth-noting that the contribution of Tollip to inflammation may be highly complex and context dependent. Excessive and robust inflammation manifested in the septic colitis model often triggers compensatory tolerance. Lack of tolerance due to Tollip deficiency further exacerbates the inflammatory pathogenesis of septic colitis. In contrast, under low-grade inflammatory conditions in which homeostatic leukocyte tolerance is not triggered, Tollip was shown to be channeled to mitochondria and perform distinct functions un-related to tolerance induction^16^. The context-dependent Tollip functions may govern resolving vs non-resolving inflammation.

Taken together, our data provide novel mechanisms as well as translational relevance involving reduced Tollip levels that underlie the programming of incompetent neutrophils in septic colitis. Future potential strategies aimed at either increasing Tollip expression and/or function may hold therapeutic promise in the treatment of severe colitis and related inflammatory complications.

## Methods

### Mice

Wild type (WT) C57BL/6 mice were purchased from the Charles River laboratory. Tollip^−/−^ mice on C57BL/6 background were provided by Dr. Jürg Tschopp in the University of Lausanne at Switzerland. All mice were housed under specific pathogen-free conditions and bred and maintained in the animal facility at Virginia Tech. Male mice with body weight: 25 ± 1 g and 7–9 weeks of age were used in all experiments. All experimental protocols were approved by the Animal Care and Use Committee (IACUC) from Virginia Tech.

### Human subjects

Peripheral blood samples were collected from six healthy volunteers and eight colitis patients from Southern Medical Hospital with informed consent from all subjects, and the research activities were approved by the Institutional Review Board (IRB) of the Southern Medical Hospital, China. For heathy controls, they should be performed physical examination recently without any disorders founded, and the age ranged from 20 to 60 years old. For the active ulcerative colitis (UC) patients, they should be performed with endoscopy and diagnosed with active inflammation in colon in one week. And they never received bio-therapeutics since they were diagnosed with UC. The age also ranged from 20 to 60 years old. All methods for both human and animal experiments were performed in accordance with the relevant guidelines and regulations as approved by the IRB and IACUC of Virginia Tech as well as Southern Medical University.

### Reagents

LPS (Escherichia coli 0111:B4), TUDCA were obtained from Sigma Aldrich. Anti-FPR2 (M-73) antibody was obtained from Santa Cruz Biotechnology. Antibodies against phospho-AKT^473^, LAMP1, phospho-CREB, phospho-MSK1, GAPDH were purchased from Cell Signaling Technology. FITC conjugated anti-mouse Ly6G antibody, PE conjugated anti-mouse CD195(CCR5) antibody, PE/Cy7 conjugated anti-mouse Ly6C antibody, APC/Cy7 and PE conjugated anti-mouse/human CD11b antibody were from Biolegend (San Diego, CA). FPRL1 (bs-3654R) antibody was from Bioss Antibodies. Anti-citrullinated Histone H4 antibodywas fromMillipore (Billerica,MA). ELISA Kit for TNF-α, IL1-β, IL-6 and IL-10 from eBioscience respectively. MPO ELISA Kit from R&D system.

### Induction of acute colitis

WT or Tollip^−/−^ mice were randomly separated to DSS group and control group: Mice of DSS group were given 3% (w/v) DSS (MW 47,000; MP Biomedicals) in drinking water ad libitum for 6 days followed by normal drinking for another 6 days; control group were given regular drinking in the whole experimental process. Mice were sacrificed at day 6 and day12 during the experiment (Fig. 1A).

### Disease activity

The disease activity of colitis was performed by clinical score as shown in Fig. S1. Weight loss, rectal bleeding and stool consistency were assessed, then averaged every day during the whole animal experimental process. In addition, colon lengths were measured for each animal at the completion of each study.

### Pathology

Colonic tissues were embedded in paraffin and stained with H&E. Histological severity of acute colitis in DSS-treated mice was determined using combined scores as reported: each sample was graded from 0 to 5 according to the score shown in Fig. S2. All histopathological evaluations were done in a blinded fashion by an independent pathologist

### Colon tissue culture and pro-inflammatory mediator assessments

Colons were washed with PBS containing penicillin/streptomycin followed the distal-most 3 cm were isolated and further cut into1–2 cm sections. Colon sections were covered with RPMI media, including 1% FBS and 1% penicillin/streptomycin, and incubated overnight. Cell-free supernatants were harvested for further ELISA assay.

### ELISA of Cytokines

Collected plasma and the supernatants of colon tissue culture were used for the measurement of TNF-α, IL1-β, IL-6 and IL-10 levels by enzyme-linked immunosorbent assay (ELISA) using ELISA Kit from eBioscience respectively. Myeloperoxidase (MPO) levels of colon culture and neutrophils culture medium were assessed using mouse MPO Elisa Kit from R&D system.

### Chemotaxis Assays

Chemotaxis of neutrophils was measured with 48-well micro-chambers and polycarbonate filters (5 μm poresize) (NeuroProbe, Cabin John, MD). The results were expressed as the Mean ± SEM of the chemotaxis index (CI), representing the fold increase in the number of migrated cells in response to chemo-attractants over spontaneous cell migration (to control medium) as previously mentioned.

### Purification and culture for bone marrow-derived neutrophil

Neutrophils from bone marrow were isolated from the tibias and femurs of WT and Tollip^−/−^ mice, and cultured with completed RPMI(10% FBS,1% penicillin/streptomycin,1% Glycine) including 10 ng/ml GM-CSF in untreated tissue culture dishes for overnight. For purification, 62.5% percoll was used to get neutrophil pellet. Cells were suspended in PBS for further experiment.

### Immunoblotting

Cells were washed with PBS and harvested in a 1 × SDS lysis buffer containing protease inhibitor cocktail and subjected to SDS-PAGE. The protein bands were transferred to an immunoblot PVDF membrane (BioRad). Western analyses were performed with specified antibodies as shown in reagents above.

### Flow cytometry

Single-cell suspensions were prepared from blood or bone marrow, and were stained with fluorescent Abs (BioLegend) in the presence of 2.4G2 (anti-FcgII/III receptor). Samples were analyzed with a FACSCanto II (BD Biosciences). FACS plots shown were analyzed with FlowJo (Ashland, OR).

For the measurement of neutrophil extracellular traps (NET) by flow cytometry,neutrophils were harvested from bone marrow, cultured, and treated with LPS (1 μg/ml) overnight. For assay of NET formation by FACS as reported^6^. Cells were fixed and permeabilized (BD Phosflow™ buffer, BD Biosciences, San Jose, CA), then labeled with anti-citrullinated Histone H4 antibody (Millipore, Billerica, MA), anti-Ly6G and anti-CD11b antibodies (BioLegend, SanDiego, CA). The samples were then analyzed by FACSCanto II (BD Biosciences). The data were processed by Flow Jo (Tree Star, Ashland, OR).

### Neutrophil bacteria killing assay

For bacteria killing assay, purified neutrophils were cultured and treated with LPS (1 μg/ml) overnight, then adjusted the cell concentration to 1 × 10^6^ /ml and mixed with 10 ul bacteria solution (1 × 10^6^/ml) for 1 hour, then 20 ul mixed solution of neutrophil/bacteria was used to smear. The smear on plates were covered with LB gel, and cultured overnight. Bacteria colony was counted next day.

### Statistics

Results were expressed as Mean ± SEM. Statistical significance between groups were determined using one-way analysis of variance (ANOVA) with Tukey’s multiple comparison test. Differences between two groups were evaluated using an unpaired two-tailed Student’s t test. p values < or = 0.05 were considered statistically significant. GraphPad Prism statistical software was used for analysis^33^.

## Additional Information

**How to cite this article**: Diao, N. *et al.* Deficiency in Toll-interacting protein (Tollip) skews inflamed yet incompetent innate leukocytes *in vivo* during DSS-induced septic colitis. *Sci. Rep.* **6**, 34672; doi: 10.1038/srep34672 (2016).

## Supplementary Material

Supplementary Information
