# Supplementary material for: Deficiency in Toll-interacting protein (Tollip) skews inflamed yet incompetent innate leukocytes in vivo during DSS-induced septic colitis
Source: Sci Rep. 2016 Oct 5;6:34672. doi: 10.1038/srep34672 (PMC5050405; doi:10.1038/srep34672)
Supplement: Supplementary Information [file srep34672-s1.pdf]

Supplementary Information

Deficiency in Toll-interacting protein (Tollip) skews inflamed yet incompetent innate leukocytes *in vivo* during DSS-induced septic colitis

Na Diao[1, 2], Keqiang Chen[1], Yao Zhang[1], Ruoxi Yuan[1], Christina Lee[1], Shuo Geng[1], Elizabeth Kowalski[1], Wen Guo[2], Huabao Xiong[3], Mingsong Li[2*], Liwu Li[1*]

1. Department of Biological Sciences, Biomedical Engineering, Medicine, Virginia Tech, USA 24061.

2. Guangdong Provincial Key Laboratory of Gastroenterology, Department of Gastroenterology, Nanfang Hospital, Southern Medical University, People's Republic of China 510515

3. Department of Medicine, Immunology Institute, Icahn School of Medicine at Mount Sinai, New York, NY 10029, USA

Running title: Tollip in Acute Colitis

Key words: Tollip; neutrophil; activation dynamics; Inflammation; colitis

*Address correspondence and reprint requests to Prof. Liwu Li, Department of Biological Sciences, Virginia Polytechnic Institute and State University, Life Science 1 Building, Washington Street, Blacksburg, VA 24061. E-mail address: lwli@vt.edu
or Dr. Mingsong Li, Guangdong Provincial Key Laboratory of Gastroenterology, Department of Gastroenterology, Nanfang Hospital, Southern Medical University, People's Republic of China 510515. E-mail address: lims@smu.edu.cn

SCORE	Body weight loss	Stool consistency	bleeding
0	No weight loss	Well formed pellets	No blood
1	Weight loss of 1-5%	Softer and well-formed stool	Positive hemoccult
2	Weight loss of 5-10%	Softer but semi-formed stool	Visible Red
3	Weight loss of 10-20%	Pasty stools that did not stick to the anus	Dark Red
4	more than 20%	Liquid stools that remained adhesive to the anus	Gross bleeding from the rectum

Supplementary Figure S1. The score system used to assess the clinical manifestation

Score	Crypt damage	PMN infiltration	Range of crypt damage and inflammation
0	Intact	Few PMN	Normal
1	Loss of the basal 1/3 of crypt	1-3 PMN/field in the submucosa	1/5 colon
2	Loss of basal 1/2 of the crypt	>3 PMN/field in the submucosa	2/5 colon
3	Loss of basal 2/3 of the crypt	1-3 PMN/field in the mucosa	3/5 colon
4	Entire loss of the crypt	>3 PMN/field in the mucosa	4/5 colon
5	Loss of crypt and surface epithelia	Numerous PMN in the epithelium	Whole colon

Supplementary Figure S2. The score system used to assess histopathological changes of gastrointestinal disease

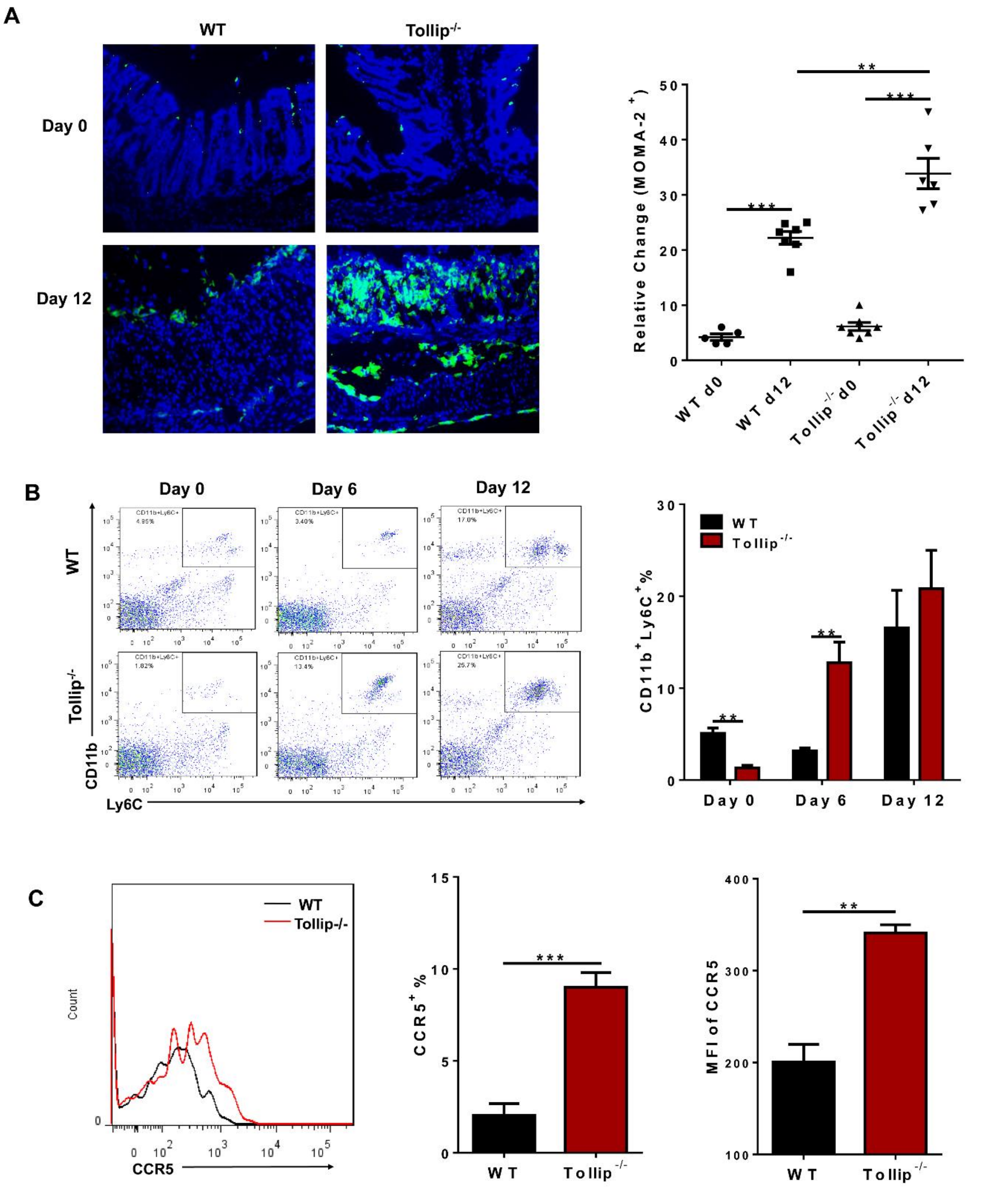

Supplementary Figure S3. Monocytes were induced both at local level and systemic level. A. Immuno-histological staining (left) of monocytes (MOMA-2$^+$) in the injured mucosa of colon.

Assessment (right) about monocytes with stained particles using Image J. **B.** Flow cytometry results of monocytes (Ly6G$^-$CD11b$^+$Ly6G$^+$) in blood harvested on day 0, day 6, day 12 during the process of DSS induced colitis. C. CCR5 expression on neutrophils in blood via Flow Cytometry. Blood was harvested on day 6 during the process of DSS induced colitis.

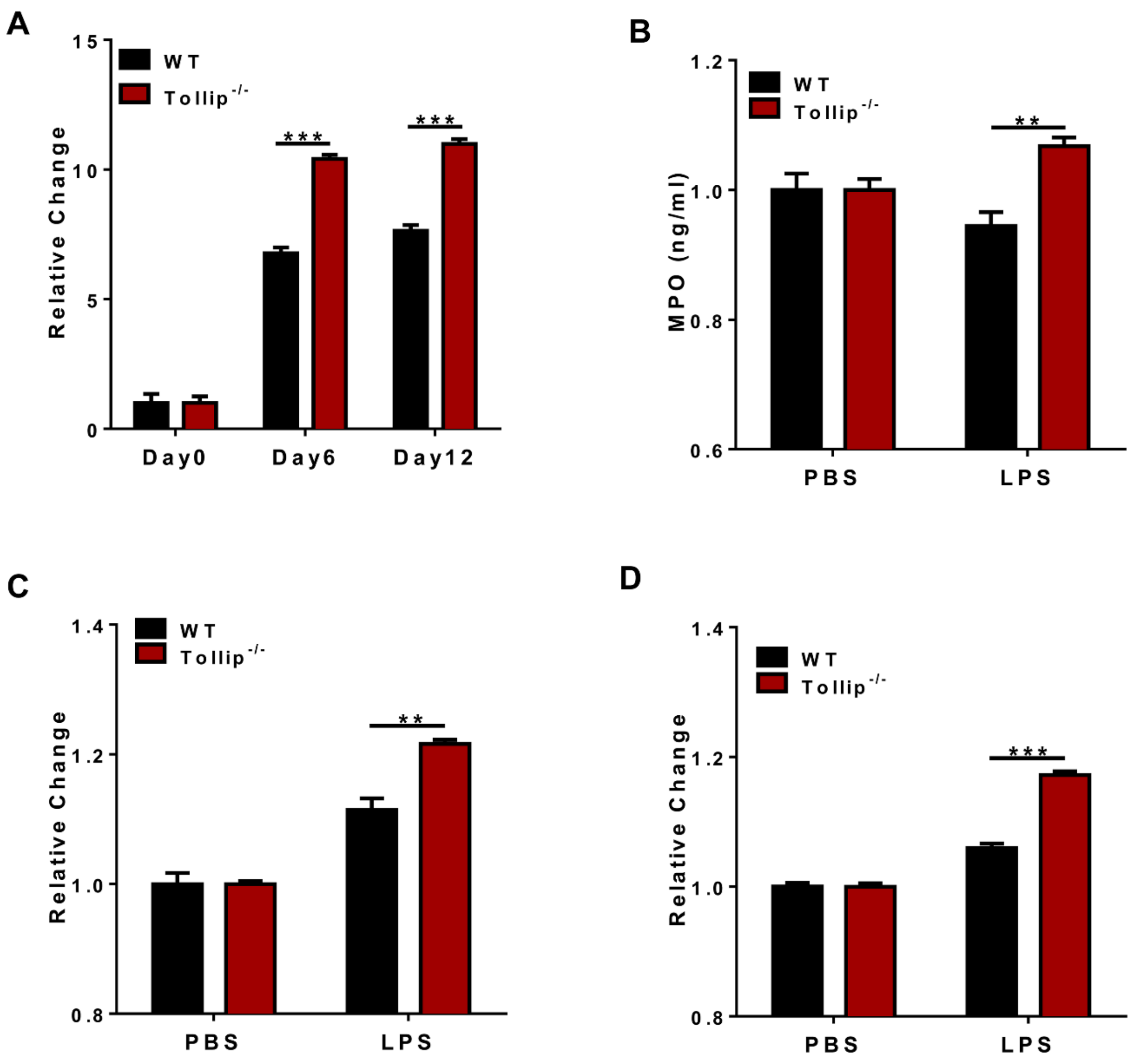

Supplementary Figure S4. The MPO production is relatively increased in Tollip$^{-/-}$ mice compared with WT both in vivo and in vitro.

A.Relative MPO level in conlon culture supernantant. B.Relative MPO production in culture medium of neutrophils isolated from peritoneal and treated with 1μg/ml of LPS for 4 hours (B) and from bone marrow and treated with 1μg/ml of LPS for 2 hours (C) and 4 hours(D). * * P < 0.01, * * * P < 0.01.

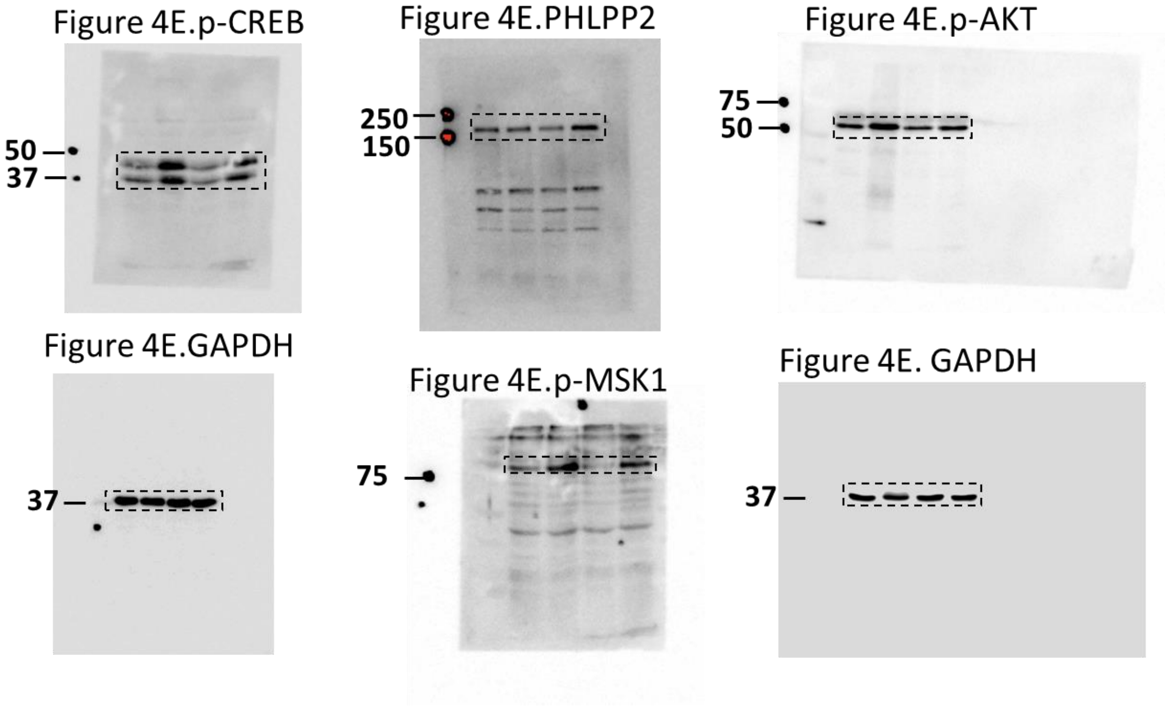

Supplementary Figure S5. Uncropped gel images with size marker indications. Dashed lines are cropping lines.
